# Review of Recent Microwave Planar Resonator-Based Sensors: Techniques of Complex Permittivity Extraction, Applications, Open Challenges and Future Research Directions

**DOI:** 10.3390/s21072267

**Published:** 2021-03-24

**Authors:** Rammah Ali Alahnomi, Zahriladha Zakaria, Zulkalnain Mohd Yussof, Ayman Abdulhadi Althuwayb, Ammar Alhegazi, Hussein Alsariera, Norhanani Abd Rahman

**Affiliations:** 1 Microwave Research Group, Centre for Telecommunication Research and Innovation (CeTRI), Faculty of Electronics and Computer Engineering, Universiti Teknikal Malaysia Melaka (UTeM), Durian Tunggal, Melaka 76100, Malaysia; alrammah89@gmail.com (R.A.A.); zulkalnain@utem.edu.my (Z.M.Y.); ammarhejazy@hotmail.com (A.A.); husseinalsareira@gmail.com (H.A.); norhanani80@gmail.com (N.A.R.); 2 Electrical Engineering Department, Jouf University, Sakaka, Aljouf 72388, Saudi Arabia; aaalthuwayb@ju.edu.sa; 3 Department of Electrical Engineering, Politeknik Port Dickson (PPD), Port Dickson, Negeri Sembilan 71250, Malaysia

**Keywords:** microwave sensor, complex permittivity extraction, biosensor application, electric field distribution, resonators

## Abstract

Recent developments in the field of microwave planar sensors have led to a renewed interest in industrial, chemical, biological and medical applications that are capable of performing real-time and non-invasive measurement of material properties. Among the plausible advantages of microwave planar sensors is that they have a compact size, a low cost and the ease of fabrication and integration compared to prevailing sensors. However, some of their main drawbacks can be considered that restrict their usage and limit the range of applications such as their sensitivity and selectivity. The development of high-sensitivity microwave planar sensors is required for highly accurate complex permittivity measurements to monitor the small variations among different material samples. Therefore, the purpose of this paper is to review recent research on the development of microwave planar sensors and further challenges of their sensitivity and selectivity. Furthermore, the techniques of the complex permittivity extraction (real and imaginary parts) are discussed based on the different approaches of mathematical models. The outcomes of this review may facilitate improvements of and an alternative solution for the enhancement of microwave planar sensors’ normalized sensitivity for material characterization, especially in biochemical and beverage industry applications.

## 1. Introduction

**Figure 1:**
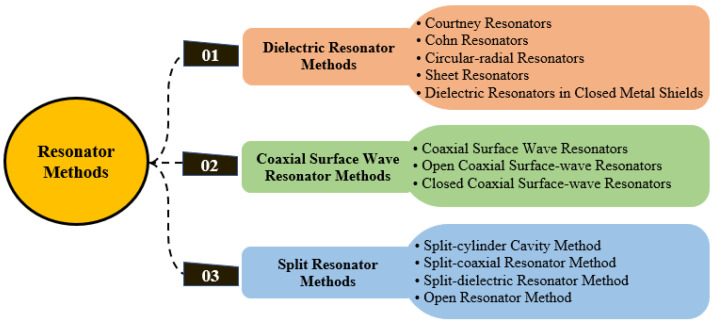
Classification methods based on resonators for the study of material properties.

By definition, microwave sensors utilize electromagnetic fields and are devices internally operating at frequencies starting from 300 MHz up to the terahertz range. Various types of microwave planar sensors have been developed over the past decade. Certain types are known, while some are new and surprising [1]. Resonator sensors are some of these types, which are configured to have a resonance frequency or relative oscillation phase dependent on the measured parameters [2]. Resonator sensors can operate either passively where the sensing principle is based on naturally emitted or reflected radiation from the object or target under observation or they may be active sensors where the sensors emit microwave radiation and then sense reflected microwaves from the object or target under observation [3] (this study restricts its coverage to passive resonator sensors). In recent years, resonator sensors have played a key role in materials’ characterization and their applications. Resonant techniques are normally given very much attention due to the high accuracy and sensitivity compared to prevailing techniques. Several techniques are new concepts, whereas others have been developed from previous research works in the literature. This compilation of research studies can lead to a change in developing and enhancing planar sensors for material properties’ characterization. Due to industrial demand, researchers have competed with each other to design techniques that have a high accuracy, sensitivity and compact size. There are many techniques available for the measurement of the real and imaginary parts of the complex permittivity (dielectric constant, loss tangent). Figure 1 demonstrates the methods of the resonator for extracting the material and dielectric properties.

Although resonators are considered for their key performance in a range of RF/microwave communications and electronics applications such as oscillators and filters, significant efforts have been devoted to microwave resonator sensors for material characterization applications. The importance of studying material characterization is that this can be used for many applications such as agriculture, which is involved in grain drying, seed treatment in order to improve seed germination and insect control in stored grain using high-frequency and microwave electric fields. In addition, this is used for food processing purposes to check the quality and safety of food. This is also used for dairy products to roughly determine the content of milk in terms of ionic compounds, fats, carbohydrates and proteins. On the other hand, this is used for fruits and vegetables because there is a need for rapid non-destructive quality measurements to know the freshness of fruits and vegetables. This is also used for geoscience because when studying the dielectric properties of the soil, this would be helpful for planting. Furthermore, this is used for biosensing in order to know the properties of tissues and cells and to increase the knowledge of biological processes at the molecular level, and this is also involved in continuing developments in radio frequency and microwave hyperthermia treatments for cancer. Finally, this is used in the pharmaceutical industry in order to check and validate the quality and safety of medicines [4,5,6,7,8].

The analysed literature was found through scientific service tools using “microwave sensor” and “microwave planar resonators” as queries. Filtered articles published from 2012 to 2020 were adopted in this research, and they were used to review the main streams of research focusing on microwave resonator sensors and their general use in material characterization. This review shows the recent development of various studies and examples of applications of microwave planar sensors in material characterization. It includes the mathematical models for complex permittivity extraction [9,10,11,12,13,14,15,16,17,18,19,20,21,22,23,24], sensing application examples of microwave sensors [25,26,27,28,29,30,31,32,33,34,35,36,37,38,39,40,41,42,43,44,45,46,47,48,49,50,51,52,53,54,55,56,57,58,59,60,61,62,63,64,65,66,67,68,69,70,71,72] and recent developments and their challenges and future directions [24,73,74,75,76,77,78,79,80,81,82,83,84,85,86,87,88,89,90,91,92,93,94,95,96,97,98,99,100,101,102,103,104,105,106,107,108,109,110,111,112,113,114,115,116,117,118,119,120,121,122,123,124,125,126,127,128,129,130,131,132,133,134,135,136,137,138,139,140,141,142,143,144,145,146,147,148,149,150,151,152,153,154,155,156,157,158,159,160,161,162,163,164,165,166,167,168,169,170,171,172,173,174]. This work is mainly focused on microwave sensor-based planar resonators for material properties’ extraction such as solids, liquids and their binary mixtures. The material properties’ extraction is in terms of the complex permittivity (real and imaginary parts), and other material properties such as the permeability and conductivity of materials are not included.

## 2. Mathematical Modelling for Complex Permittivity Extraction

Various numerical methods have been used for extracting the complex permittivity or dielectric properties of materials such as the Nicholson–Ross–Weir (NRW), NIST iterative, new non-iterative, the Rational Function Model (RFM), and the frequency and quality factors methods. In most cases, it is important to know the appropriate measurement technique and conversion methods for a material in order to measure its dielectric properties. Table 1 demonstrates a general overview of various measurements techniques and conversion methods along with their advantages and disadvantages [175,176,177]. In this section, the main focus is based on resonant methods and their conversion methods for extracting the dielectric properties of materials in terms of the complex permittivity.

### 2.1. Polynomial Curve Fitting Model

The resonance frequency is dependent on the relative permittivity of tested materials [9]. A numerical model is required to characterize the permittivity of the tested materials from the measured parameters such as a change in resonance frequency and insertion loss when the sensor is loaded with the material. Using the curve fitting technique, the equation for the material permittivity is extracted from the measured scattering data, and the permittivity of the sample is mathematically expressed in terms of the curve fitting technique such as linear or polynomial. To determine the relative permittivity of the tested sample, an empirical equation can be modelled based on the relationship between the frequency ($f_{o}$) and the permittivity ($\epsilon_{r}$) using the curve fitting method, which is the linear curve fitting as expressed below:

(1) $$\epsilon_{r}=af_{o}+b$$

The polynomial curve fitting can be used to extract the permittivity of the tested material. A common polynomial model is generally used based on the second order polynomial, as indicated in the expression below:

(2) $$\epsilon_{r}=af_{o}^{2}+bf_{o}+c$$

where the coefficients a,b,c can be found by using standard Materials Under Test (MUTs).

### 2.2. The Least Squares Model Based on Peak Attenuation

To develop a mathematical sensing model for the sensor, the measurements of the resonance frequency and the peak attenuation, which are related to complex permittivity (real and imaginary parts), are required based on a binary mixture of aqueous solutions [10,11,12,13]. The measurements of the binary mixture are firstly used for calibration in order to determine the properties of the tested mixture of liquid materials in terms of the complex permittivity ($\epsilon^{\prime }$ + j$\epsilon^{\prime \prime }$). To this aim, a derivation of the mathematical relation will be used based on shifting frequency and peak attenuation with respect to the complex permittivity of the tested binary mixture materials (such as: water–ethanol and water-methanol solutions). The change of frequency (Δf) is defined as the difference between the resonance frequency when the sensor is loaded with the tested sample and the reference resonance frequency, while the change of peak attenuation ($\Delta |S_{21}$) is defined as the difference between the peak attenuation when the sensor is loaded with the tested sample and the reference peak attenuation. In the circumstance that the binary mixture is water-ethanol or water-methanol with a variation from 0% to 100% with a step size of 10%, this will produce 11 test datasets for developing the mathematical model based on nonlinear least squares fitting. The advantage of using this model is that the tolerances of the fabricated device are fully taken into account. The curve fitting of the nonlinear least squares will be used to derive an equation that describes the behaviour response of the change in the resonance frequency and peak attenuation with respect to the change of the complex permittivity as follows [10,11,12,13]:

(3) $$\left[\begin{array}{c} \Delta f_{o}\\ \Delta |S_{21}| \end{array}\right]=\left[\begin{array}{cc} m_{11} & m_{12}\\ m_{21} & m_{22} \end{array}\right]\left[\begin{array}{c} \Delta \epsilon^{\prime }\\ \Delta \epsilon^{\prime \prime } \end{array}\right]$$

where $\Delta \epsilon_{sam}^{\prime }$ = $\epsilon_{sam}^{\prime }-\epsilon_{ref}^{\prime }$, $\Delta \epsilon_{sam}^{\prime \prime }$ = $\epsilon_{sam}^{\prime \prime }-\epsilon_{ref}^{\prime \prime }$, $\Delta f_{sam}$ = $f_{sam}-f_{ref}$ and $\Delta |S_{21}|$ = $|S_{21}{|}_{sam}-{\left|S_{21}\right|}_{ref}$, with subscript sam for the sample and ref for the reference mixture. The values for $|S_{21}{|}_{sam}$ and $|S_{21}{|}_{ref}$ in the matrix are determined as $f_{sam}$ and $f_{ref}$, respectively.

In order to determine the unknown coefficients of the model in Equation (2), the datasets of the tested binary mixture will be used. The least squares method, which was explained by Ebrahimi et al. [11,12], can be used to approximate the unknown coefficients. Three matrices are required to approximate the unknown coefficients, which were extracted from the reported complex permittivity by Bao, Swicord and Davis [14], the measured resonance frequency and the measured $S_{21}$ as:

(4) $$X=\left[\begin{array}{cc} \Delta \epsilon_{sam1}^{\prime } & \Delta \epsilon_{sam1}^{\prime \prime }\\ \Delta \epsilon_{sam2}^{\prime } & \Delta \epsilon_{sam2}^{\prime \prime }\\ \Delta \epsilon_{sam3}^{\prime } & \Delta \epsilon_{sam3}^{\prime \prime }\\ \Delta \epsilon_{sam4}^{\prime } & \Delta \epsilon_{sam4}^{\prime \prime }\\ \vdots & \vdots \\ \Delta \epsilon_{sam11}^{\prime } & \Delta \epsilon_{sam11}^{\prime \prime } \end{array}\right],Y_{1}=\left[\begin{array}{c} \Delta f_{1}\\ \Delta f_{2}\\ \Delta f_{3}\\ \Delta f_{4}\\ \vdots \\ \Delta f_{11} \end{array}\right],\;\mathrm{and}\cdots Y_{2}=\left[\begin{array}{c} \Delta |S_{21}{|}_{sam1}\\ \Delta |S_{21}{|}_{sam2}\\ \Delta |S_{21}{|}_{sam3}\\ \Delta |S_{21}{|}_{sam4}\\ \vdots \\ \Delta |S_{21}{|}_{sam11} \end{array}\right]$$

The unknown coefficients can be calculated from:

(5) $${\left[\begin{array}{cc} m_{11} & m_{12} \end{array}\right]}^{T}={\left(X^{T}X\right)}^{-1}X^{T}Y_{1}$$

(6) $${\left[\begin{array}{cc} m_{21} & m_{22} \end{array}\right]}^{T}={\left(X^{T}X\right)}^{-1}X^{T}Y_{2}$$

### 2.3. The Least Squares Model Based on the Quality Factor

As reported in [15,16,17], the complex permittivity is dependent on both the resonance frequency and the quality factor of the sample. A simplified model is used to represent the small variation in the permittivity with respect to the change of the resonance frequency and the Q-factor. The change of the Q-factor (ΔQ) is defined as the differences between the Q-factor when the sensor is loaded with the tested sample and the reference Q-factor, which can be formed in a matrix [16]:

(7) $$\left[\begin{array}{c} \Delta f_{o}\\ \Delta |Q| \end{array}\right]=\left[\begin{array}{cc} m_{11} & m_{12}\\ m_{21} & m_{22} \end{array}\right]\left[\begin{array}{c} \Delta \epsilon^{\prime }\\ \Delta \epsilon^{\prime \prime } \end{array}\right]$$

where $\Delta \epsilon^{\prime }$ = $\Delta \epsilon_{sam}^{\prime }-\Delta \epsilon_{ref}^{\prime }$, $\Delta \epsilon^{\prime \prime }$ = $\Delta \epsilon_{sam}^{\prime \prime }-\Delta \epsilon_{ref}^{\prime \prime }$, $\Delta f_{o}$ = $f_{sam}-f_{ref}$ and Δ|Q| = ${\Delta |Q|}_{sam}-\Delta {\left|Q\right|}_{ref}$, with subscript sam for sample and ref for the reference mixture. The values for ${\left|Q\right|}_{sam}$ and ${\left|Q\right|}_{ref}$ in the matrix are determined as $f_{sam}$ and $f_{ref}$, respectively.

Similar to the previous section, the unknown coefficients of the model in Equation (6) can be overdetermined by the datasets of the tested binary mixture, and the least squares method, which was explained by Withayachumnankul et al. [16], can be used to approximate the unknown coefficients. In this case, three matrices can be set up from the reported complex permittivity by Bao, Swicord and Davis [14], the measured resonance frequency, and the measured quality factor as:

(8) $$X=\left[\begin{array}{cc} \Delta \epsilon_{sam1}^{\prime } & \Delta \epsilon_{sam1}^{\prime \prime }\\ \Delta \epsilon_{sam2}^{\prime } & \Delta \epsilon_{sam2}^{\prime \prime }\\ \Delta \epsilon_{sam3}^{\prime } & \Delta \epsilon_{sam3}^{\prime \prime }\\ \Delta \epsilon_{sam4}^{\prime } & \Delta \epsilon_{sam4}^{\prime \prime }\\ \vdots & \vdots \\ \Delta \epsilon_{sam11}^{\prime } & \Delta \epsilon_{sam11}^{\prime \prime } \end{array}\right],Y_{1}=\left[\begin{array}{c} \Delta f_{1}\\ \Delta f_{2}\\ \Delta f_{3}\\ \Delta f_{4}\\ \vdots \\ \Delta f_{1}1 \end{array}\right],\;\mathrm{and}\cdots Y_{2}=\left[\begin{array}{c} {\Delta |Q|}_{sam1}\\ {\Delta |Q|}_{sam2}\\ {\Delta |Q|}_{sam3}\\ {\Delta |Q|}_{sam4}\\ \vdots \\ {\Delta |Q|}_{sam11} \end{array}\right]$$

### 2.4. Debye Relaxation Equation

Polar liquids will be penetrated by the electric field of the microwave sensor, which leads to the rotation of the molecule and causes the loss of energy. The response of the designed sensor will be changed in terms of the resonance frequency and quality factor. These behaviours provide information in terms of the real and imaginary parts of the complex permittivity of the tested polar liquids. The complex permittivity of the polar liquids can be formed as ϵ = $\epsilon^{\prime }\left(\omega \right)-j\epsilon^{\prime \prime }\left(\omega \right)$, which is described by the Debye theory as the real and imaginary parts of the complex permittivity. The Debye relaxation model of DI water can be found in the literature [16,18,19]:

(9) $$\epsilon \left(\omega \right)=\epsilon_{o}^{\prime }\left(\omega \right)-j\epsilon_{o}^{\prime \prime }\left(\omega \right)=\epsilon_{\infty }+\frac{\epsilon_{s}-\epsilon_{\infty }}{1+j\omega \tau }$$

(10) $$\epsilon_{o}^{\prime }\left(\omega \right)=\epsilon_{\infty }+\frac{\epsilon_{s}-\epsilon_{\infty }}{1+\omega^{2}\tau^{2}}$$

(11) $$\epsilon_{o}^{\prime \prime }\left(\omega \right)=\omega \tau \frac{\epsilon_{s}-\epsilon_{\infty }}{1+\omega^{2}\tau^{2}}$$

where $\epsilon_{\infty }$ is the permittivity in the high-frequency limit, $\epsilon_{s}$ is the static, low-frequency permittivity, τ is the characteristic relaxation time, $\epsilon_{o}^{\prime }\left(\omega \right)$ is the real part of the complex permittivity and $\epsilon_{o}^{\prime \prime }\left(\omega \right)$ is the imaginary part of the complex permittivity.

### 2.5. Machine Learning Methods

**Figure 2:**
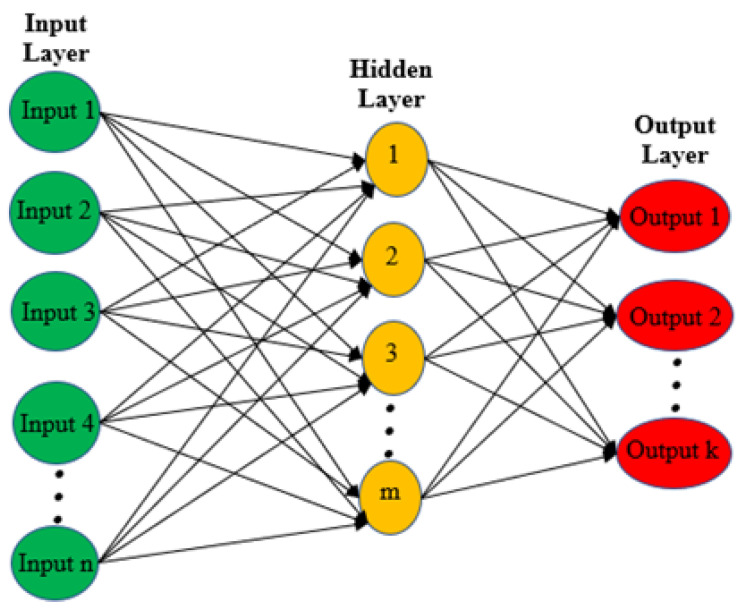
The general structure of the neural network.

Further research should be undertaken to investigate the mathematical modelling based on machine learning approaches to analyse the measured data quickly and with high accuracy. Recently, mathematical tools based on Neural Networks (NNs) were employed for complex permittivity extraction, and this is due to their strong learning ability and high accuracy [20,21,22,23,24]. Figure 2 demonstrates the general structure of a neural network where there are three layers. The first layer is the input information such as resonance frequency, quality factor and peak attenuation. The second layer is called the hidden layer, which performs the mathematical computation of thevariation on the input information and learns the patterns. The final layer is where we obtain the result performed by the hidden layer. A Back-Propagation Neural Network (BP-NN) was presented in [23] where the genetic algorithm was used to obtain the initial optimal values of the network weights in the training process, while the BP-NN was used to find the optimum solutions with these initial weights. The authors used the input data as the relative permittivity of the tested sample and the normalized quality factor. On the other hand, the output was used for the loss tangent. However, this model has drawbacks, which can be seen from this study as the BP-NN model was trained with only simulated data, and it could be better to train it with measured data to achieve higher accuracy. Another limiting factor for this model is that it requires a large amount of data and a long computational time, which affects its performance.

Another study was done by [24], where the authors presented the Fuzzy Neural Network (FNN), and they used the response behaviour of the sensor as the input layer such as the amplitude, the resonance frequency and the quality factor. These three inputs are transferred to the targeted single output, which represents the tested sample. The main advantages of this model are that there are several inputs contributed to the specific classification problem instead of only one signature. Furthermore, the performance accuracy was high because the analysis and investigation were done based on experimental data. An Artificial Neural Network (ANN) was presented by [22] to model the response behaviour of the sensor and eliminate the uncertainty. The authors developed the ANN to avoid the errors of temperature variation in fluidic characterisation. The main advantage of this technique is its ability to omit the error of the temperature effect from both the sensor and tested materials. The total achieved accuracy from the developed neural network was 92% for all the test data of all classes, which is a significantly high value. This indicates that neural models have a high accuracy and strong learning ability in a short time compared to empirical models. This proves that microwave sensors are good to employ in various environments in many important applications when equipped with the ANN system. The technique is good in various environments for many important applications.

## 3. Sensing Application Example of Microwave Sensors

### 3.1. Detection of Solid Materials

Microwave sensors based on microwave resonator sensors have been extensively reported by researchers for detecting and characterizing various solid materials operating at different frequencies. Some are based on Microstrip Ring Resonators (MRRs) [25,26,27,28,29,30,31,32,178], while others are based on Split-Ring Resonators (SRRs) [33,34,35,36,37] incorporated with Complementary Split Ring Resonators (CSRRs) [9,38,39,40,41,42,43,44]. The authors of [37,45,178] designed a sensor for detecting soil types and moisture. The error found in detecting these types of materials was 5%, which was reported by Then et al. [178] using a microstrip ring resonator at a 3.2 GHz operating frequency. Another type of application for detecting solid materials was reported by Chakyar et al. [33] using a split ring resonator to detect types of flour at a 3.57 GHz operating frequency. Prior to testing the flour samples, the authors calibrated the sensor with different materials under test, which were: plastic, Perspex, glass epoxy and glass. Solid samples of Teflon, Viz, PVC, rubber and wood were tested and reported in the literature using different techniques [9,34,38,44]. However, the maximum error was reported by Shafi et al. [34], who found it to be less than 8%. Resonator sensors are designed for detecting the meat and fat content [25,26,28,31,32,45]. The maximum discrepancy was 6%, which is found by Jilani et al. [25] using a microstrip ring resonator at a 1 GHz operating frequency. Table 2 describes the selected published articles that detected and characterised various solid materials. The comparisons are in terms of the technique, the materials for fabrication used, the operating frequency, the cross-section size, tested materials (MUTs), the application and remarks.

### 3.2. Detection of Liquid Materials

Recent developments in the field of microwave resonator sensors have led to a renewed interest in material characterization especially for liquid detection and their mixtures. Early examples of microwave resonator sensors for liquid detection include oil permittivity determination [4,47,48,49,50,51,52,53]. A non-destructive technique based on a planar RF sensor was presented by Shafi, Jha and Akhtar [47] for detecting edible oils such as: olive, coconut, soya bean, sunflower, and mustard oils. The authors also investigated the adulteration of edible oils by mixing a percentage of mustard and sunflower oils in olive oil. The ability of the sensor to detect the adulterated oils was observed since the permittivity decreased when increasing the percentage of adulteration. Furthermore, some researchers focused on detecting and characterizing petroleum oils such as petrol and diesel [4,51]. These studies are limited to a certain percentage of detection, where Kulkarni and Joshi [4] showed that the sensor could detect low permittivity materials. Table 3 demonstrates the early examples of the microwave resonators that were used for edible oil and fuel oil detection.

One of the most significant current discussions in liquid detection using microwave resonator sensors is the binary mixture of two liquid materials. Some researchers tested various combinations of water-ethanol [11,54,55], water-acetone [56] and alcohol [57], while others tested different pure liquids such as water, ethanol, methanol [11,45,54,55,56,57,58,59,60,61], acetone [56,60], petrol [62], isopropanol [60], benzene, ethyl acetate, hexane, pentane [63], chloroform [55,64], soya, lemon tea, Nescafe and Kickapoo [58]. Table 3 demonstrates various liquids’ detection based on existing studies found in the literature.

### 3.3. Detection of Glucose Concentration

Considerable studies based on microwave sensors have been done for glucose monitoring. This is due to the capability of microwave sensors to non-destructively measure the parameters inside the volume since the daily monitoring techniques of glucose are invasive, requiring a blood sample to measure the parameters [65,66,67,68]. Qiang, Wang and Kim [67] designed a biosensor combined with a volume-fixed structure to detect the level of glucose. A range of glucose concentrations was used in this study starting from 50 to 600 mg/dL, which was dropped into the volume-fixed structure for detection. The results showed a sensitivity of 1.13 MHz and 1.97 MHz per mg/dL; however, the sensor design was complicated, requiring multiple layers for manufacturing the sensor. Furthermore, the sensor offered a very small size in terms of the circuit, since it was operating at very high frequencies of 17.25 GHz and 21.09 GHz. Another split ring resonator integrated with an antenna was designed by Verma et al. [69] for sensing glucose in blood plasma. There was a change in the frequency response of the designed sensor in this study where the variations of the glucose concentration in blood plasma were used (85 mg/dL, 130 mg/dL, 150 mg/dL). This indicates that microwave resonator sensors have the capability to detect the glucose concentration level noninvasively. Table 4 demonstrates different designs that are used for glucose concentration detection.

## 4. Recent Developments of Microwave Planar Resonators

**Figure 3:**
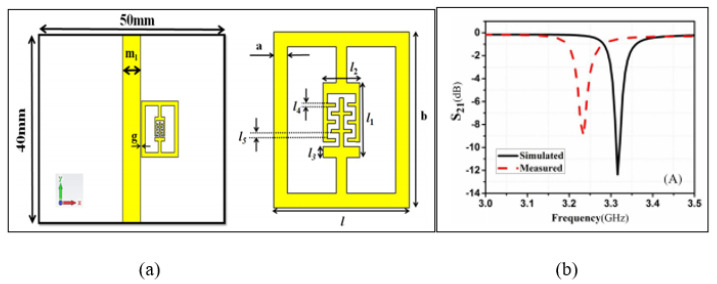
Electric-LC(ELC)-based resonator sensor: (**a**) Layout of a typical ELC sensor. (**b**) Measured and simulated transmission response of the structure [73]. Reprinted from Akhtar, M.J.; Varshney, P.K.; Kapoor, A. Interdigital capacitor loaded electric-LC resonator for dielectric characterization. *Microwave and Optical Technology Letters* 2020 with permission from John Wiley and Sons.

There is an increasing interest in developing planar resonator sensors that lead to high sensitivity and accuracy. These advantages are used to sense the properties of materials such as solid, powder and aqueous solutions and impurities including the glucose concentration in water solutions based on the complex permittivity extraction. Recently, a high sensitivity microstrip line with an Electric-LC(ELC) resonator sensor was proposed by Kapoor, Varshney and Akhtar [73]. The authors demonstrated the high sensitivity by loading the ELC element with the Interdigital Capacitor (IDC). The IDC provides an enhancement of the electric field distribution on the sensing area at the designed operating frequency of 3.316 GHz. This enhancement indicates a high sensitivity of the sensor, which essentially demonstrates its capability for the detection of small changes in the complex permittivity of the tested sample. Figure 3 illustrates the ELC resonator aligned with the patch on the front side of the substrate. In this condition, the ELC resonator will be excited by the electric field generated by the IDC and produce a sharp notch appearing at a resonance frequency of 3.316 GHz. When the ELC sensor is loaded with a sample having a permittivity of 10, the resonance frequency is shifted downwards by 1.536 GHz, forming a high sensitivity and normalized sensitivity of 170.67 MHz and 5.14%, respectively. However, these achievements are limited to the material sample, which has a permittivity range of one to 10.

**Figure 4:**
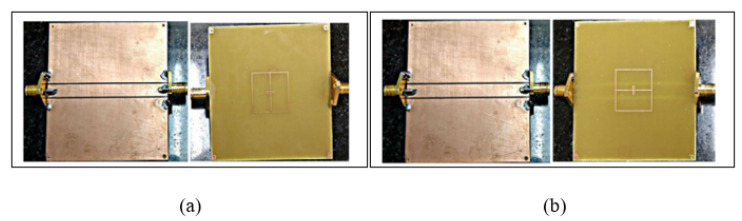
Coplanar Waveguide (CPW)-loaded ELC sensor: (**a**) First configuration. (**b**) Second configuration with rotation of the ELC resonator structure by 90° [74]. Reprinted from Akhtar, M.J.; Sharma, A.; Varshney, P.K. Exploration of adulteration in some food materials using high-sensitivity configuration of electric-LC resonator sensor. *International Journal of RF and Microwave Computer-Aided Engineering*, 2019, with permission from John Wiley and Sons.

One possibility for designing the ELC is by using the Coplanar Waveguide technology (CPW) as proposed by Varshney, Sharma and Akhtar [74]. The development was done by deploying an ELC sensor with a coupled CPW, and two design configurations were proposed by simply rotating the ELC resonator structure on the back side by 90°, which were operating at two different resonance frequencies of 3.456 GHz and 3.848 GHz for the first and second configurations, respectively. These two configurations are demonstrated in Figure 4, where the ELC aligned with the coplanar waveguide is shown and creates magnetic and electric walls. Both sensors provide a high sensitivity due to the high electric field distribution enhanced by the ELC capacitive region. However, the CPW, which is aligned with the magnetic wall of the ELC sensor, exhibits a higher sensitivity of 136 MHz. This sensor is used for exploring the adulteration of some foods such as wheat flour and chickpea flour by putting small microgram samples on top of the sensor. This study showed a good linearity among the change in resonance frequency and the adulteration percentage, comprised of a mixture of both flours in a ratio of 1:3, 2:2 and 3:1 by weight. However, the results may be affected since the sample was placed on the top of the sensor by a guided glass tube, which was then removed. It would be better if there were a holder or container to hold the sample for greater accuracy and consistency of the measurement results.

**Figure 5:**
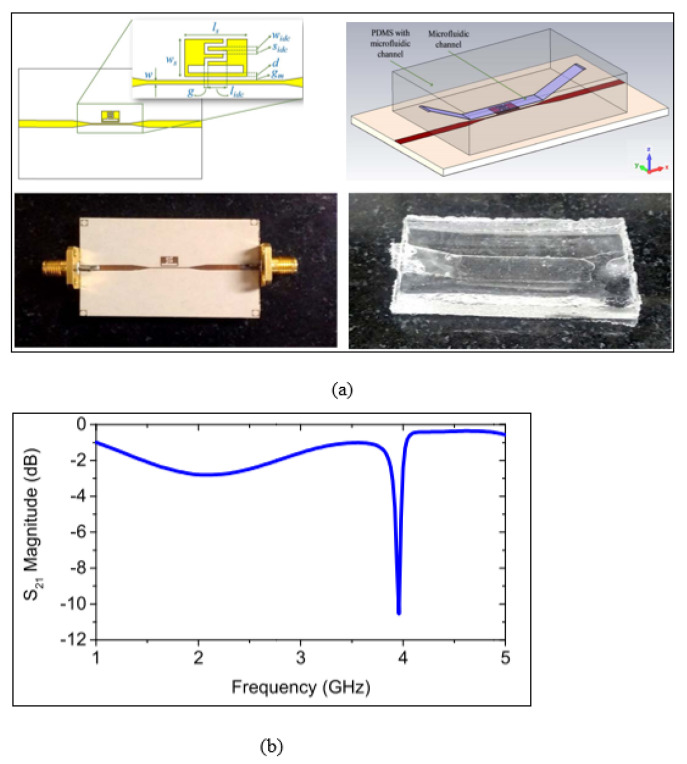
Metamaterial microwave split ring resonator sensor incorporated with an IDC: (**a**) Layout of the split ring resonator with the IDC along with the PDMS microfluidic channel. (**b**) Measured transmission response of the unloaded sensor [83]. Reprinted from Govind, G. Metamaterial-Inspired Microwave Microfluidic Sensor for Glucose Monitoring in Aqueous Solutions. *IEEE Sensors Journal*, 2019, with permission from IEEE.

Since the interdigital capacitor enhanced the electric field distribution as discussed by Kapoor, Varshney and Akhtar [73], the SRR can be incorporated with an IDC, as presented by Govind and Akhtar [83]. Figure 5 demonstrates the split ring resonator with the IDC aligned with the PDMS microfluidic channel for liquid testing. The sensor was used for monitoring glucose in aqueous solutions, and it achieved a sensitivity of 2.60 × $10^{-2}$ MHz/mg dL${}^{-1}$ at an operating frequency of 4.18 GHz. Different glucose concentrations were tested from 0 to 5000 mg/dL; however, the step size was considered very large for this study; the glucose concentrations were prepared at 0 mg/dL, 1250 mg/dL, 2083 mg/dL, 3571 mg/dL and 5000 mg/dL, which limits the capability of detecting the glucose at low concentrations.

**Figure 6:**
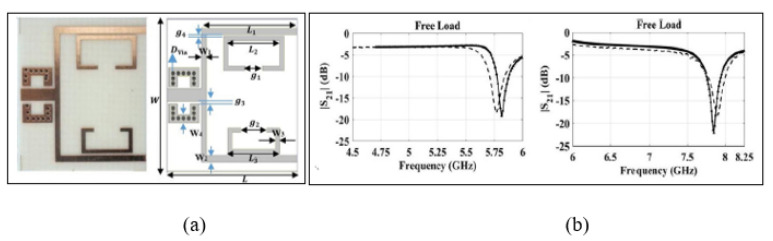
Non-Identical SRR (NID-SRR) sensor: (**a**) Layout of the NID-SRR sensor. (**b**) Measured and simulated transmission response on the dual-band NID-SRR [75]. Reprinted from Kiani, S.; Rezaei, P.; Navaei, M. Dual-sensing and dual-frequency microwave SRR sensor for liquid samples permittivity detection. *Measurement*, 2020, with permission from Elsevier.

To demonstrate a high accuracy, Kiani, Rezaei and Navaei [75] designed a dual-sensing at dual frequencies-based SRR sensor for liquid sample permittivity detection. This sensor has the capability of measuring one tested sample using two frequency bands, which decreases the measurement error caused by a single frequency. Not only that, it also can perform dual sensing for two tested samples at the same time. The developed technology is based on non-identical SRR sensors, which are placed inside power divider branches. Figure 6 demonstrates a Non-Identical Double-SRR (NID-SRR) sensor, which is used to test several samples simultaneously. The sensor exhibits a sensitivity of 16 MHz and 22 MHz for SRR1 and SRR2, respectively. It also exhibits a normalized sensitivity of 0.28 and 0.3 for SRR1 and SRR2, respectively, and it can perform testing of materials that have properties in the range of 24 to 78 relative permittivity. Another advantage of this developed sensor is that it can use one band as the testing sample and another band as the reference, which will monitor the effects of surrounding environment factors such as ambient temperature. However, the problem of this type of resonator is the mutual influence of the channels, which requires using a powder divider to reduce it.

**Figure 7:**
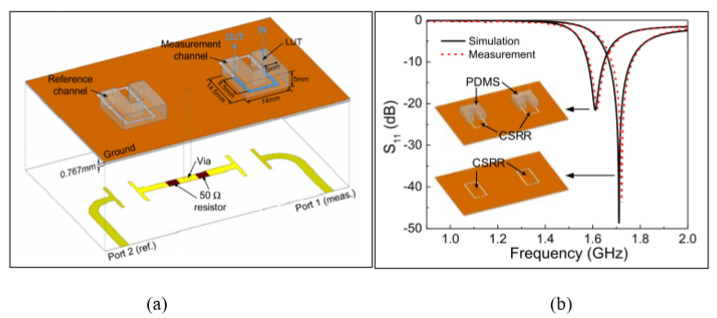
The microstrip CSRR-based sensor: (**a**) Layout of the MCSRR-based sensor. (**b**) Measured and simulated reflection response with and without the microfluidic PDMS channels [23]. Reprinted from Gan, H.Y. Differential Microwave Microfluidic Sensor Based on Microstrip Complementary Split-Ring Resonator (MCSRR) Structure. *IEEE Sensors Journal*, 2020, with permission from IEEE.

Similarly, the CSRR-based differential microwave sensor can be used for dual-sensing, which was implemented in microstrip technology, as presented by Gan et al. [23]. Figure 7 demonstrates the differential microwave sensor based on the microstrip CSRR structure aligned with microstrip feedlines patched on the back side of the substrate. These microstrip feedlines were mounted by virtue of two 50 Ohm resistors among the corresponding two SRRs for differential sensing. The electric field of the CSRR sensor is mainly distributed and focused along the slot of the CSRR on the metal of the ground plane. This leads to a high normalized sensitivity of 0.626% for a permittivity range from one to 80 with the capability of environmental factors’ suppression, such as humidity and temperature. Two PDMS channels were used, where one was considered as the reference channel and the other one as the measurement channel for the liquid under test. However, the proposed sensor by Gan et al. [23] requires two resistors to be soldered, which increases the fabrication cost and circuit size and makes it inconvenient. Furthermore, the resonance of the reference channel might be affected to some extent due to the timing difference when loading the liquid sample compared to the measurement channel.

**Figure 8:**
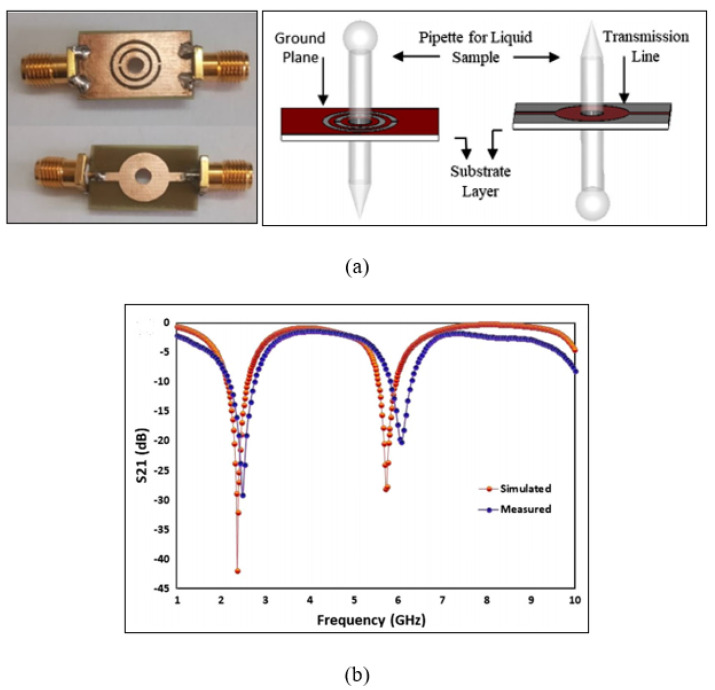
Microwave sensor based on a complementary split ring resonator: (**a**) Layout of the CSRR sensor along with the pipette for liquid samples. (**b**) Measured and simulated transmission response for a bare sensor [82]. Reprinted from Khanna, Y. et al. Dual-Band Microwave Sensor for Investigation of Liquid Impurity Concentration Using a Metamaterial Complementary Split-Ring Resonator. *Journal of Electronic Materials*, 2019, with permission from Springer Nature.

In the same vein, Khanna and Awasthi [82] presented a dual-band microwave sensor based on a CSRR operating at 2.45 GHz and 5.8 GHz. The CSRR was etched on the ground of the substrate, which induced a magnetic field due to inductance and generated the appropriate capacitance. This combination of inductance and capacitance concentrated the field distribution on the ground plane. Figure 8 indicates the CSRR for both the ground and transmission line layers aligned with a hole for the pipette to test liquid materials. The sensor is contactless and has a fixed sample position for testing materials such as milk-urea solution and water-ethanol concentration. When filling the pipette with a liquid sample, the responses of both dual-bands change. This indicates that the sensor was not specifically designed to evaluate dual-sensing related to dual frequencies where, many researchers used one band as a reference sample and the other band for sensing samples [23,75].

**Figure 9:**
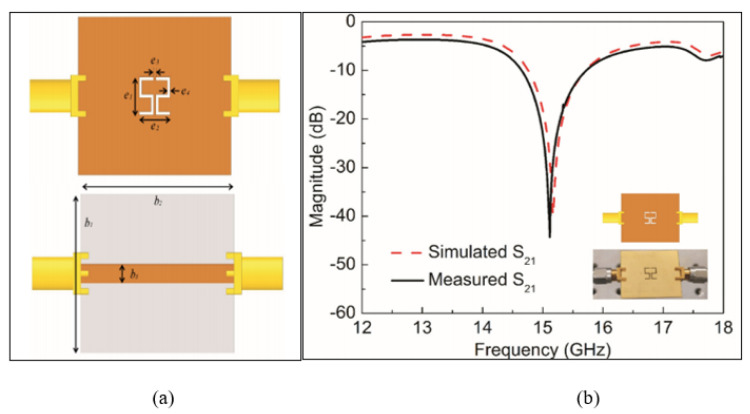
The Complementary Symmetric S-Shaped Resonator (CSSSR): (**a**) Layout of the CSSSR sensor. (**b**) Measured and simulated transmission response. The complementary SSSR is etched out of the ground plane metallisation in the bottom view and excited by the feedline in the top view [76].

Conversely, the CSRR can be integrated with the design of the metamaterial transmission lines for single-sensing. Haq et al. [76] proposed a new Complementary sensor based on a Symmetric S-Shaped Resonator (CSSSR), which is a negative image of an S-shaped resonator. The CSSSR was etched out of the ground plane metallisation and excited by the feedline in the top view. Figure 9 illustrates the design of the CSSSR sensor at a high operating frequency of 15.12 GHz. The CSSSR design provides differential sensitivity by varying the relative permittivity of tested materials. The electric field at the resonance frequency is mainly concentrated at the CSSSR edges, which elucidates the sensing area of the sensor. The sensor exhibits a high normalized sensitivity of 6.7% at the resonance frequency. However, the sensor can be used for low permittivity materials as determined by a range from 2.1 to three, which limits its capability to detect high permittivity materials and it applications.

**Figure 10:**
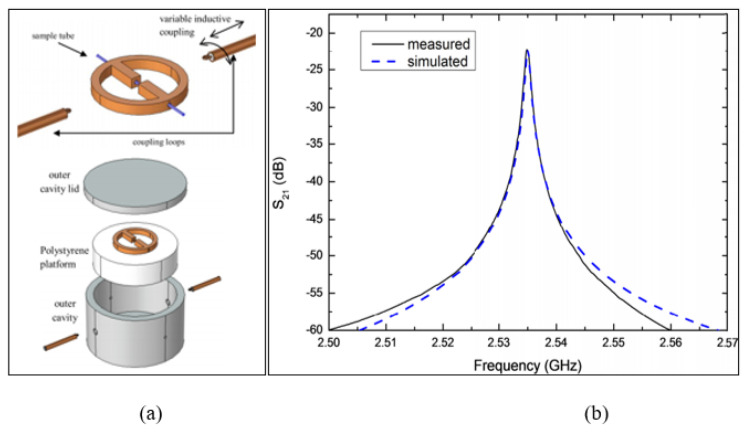
Central Gap Ring Resonator (CGRR) sensor: (**a**) Layout of the CGRR sensor including the CGRR, variable inductive coupling feedlines, outer aluminium, polystyrene platform, and PTFE sample tube. (**b**) Measured and simulated transmission response with an empty PTFE tube [77]. Reprinted from Hamzah, H. High *Q* Microwave Microfluidic Sensor Using a Central Gap Ring Resonator. *IEEE Transactions on Microwave Theory and Techniques*, 2020, with permission from IEEE.

A new type of resonator was produced by Hamzah, Abduljabar and Porch [77], which is based on the central gap resonator aligned with the variable inductive coupling of feedlines. It was modelled by an equivalent parallel LC circuit where the Central Gap Ring Resonator (CGRR) inductive regions (L) were formed by ring circumferential regions. In this case, the magnetic field was at its maximum, which was then coupled using coupling loops of inductive input-output. The sensitivity was maximized by making the tube of liquid in a parallel position to the central gap electric field distribution, which minimized the depolarization effect. Figure 10 illustrates the design of the CGRR, operating at a 2.5 GHz resonance frequency. The CGRR sensor achieved a high quality factor (Q≈3000) for detecting the relative permittivity of both new and damaged oil samples. However, this CGRR sensor was fixed on a polystyrene dielectric with a very large diameter and a height of 70 and 18 mm, respectively. Then, it was placed inside an aluminium tubewith a very large inner diameter and depth of 70 and 37 mm, respectively, which increased the fabrication and measurement cost and the circuit size, making it inconvenient.

**Figure 11:**
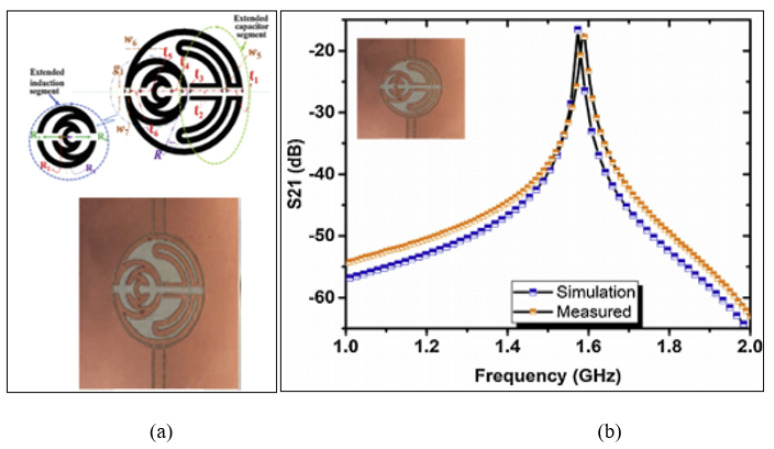
Miniaturized coplanar waveguide SRR (MSRR) sensor: (**a**) Layout of the MSRR sensor including an extended inductive and capacitive segment. (**b**) Measured and simulated transmission response in the unloaded condition [78]. Reprinted from Hosseini, N,; Olokede, S.S.; Daneshmand, M. A novel miniaturized asymmetric CPW split ring resonator with extended field distribution pattern for sensing applications. *Sensors and Actuators A: Physical*, 2020, with permission from Elsevier.

One possible design for a split ring resonator is to use a CPW. A new technique was proposed by Hosseini, Olokede and Daneshmand [78] based on a Miniaturized coplanar waveguide SRR (MSRR) operating at a 1.57 GHz frequency. This technique was developed by the integration of a half-wavelength conductor and the extended capacitive coupling gap. Figure 11 illustrates the design of the miniaturized coplanar waveguide SRR aligned with the fractal shape with extended inductive and capacitive segments. It creates a wider concentrated field area by turning the half-wavelength conductor into a fractal shape to create a circular ring enclosing. Thus, the authors extended the inductive segment and capacitive segment to produce a wider sensing area, suitable for large testing samples. However, the electric field distribution was expended on the sensor and produced a low E-field of 5 × $10^{3}$ (v/m), which limited the sensitivity of detecting the permittivity of the materials. While using a small testing sample, the E-field should be concentrated in a certain area that fits the tested sample since the MUT will absorb the energy by the perturbation theory and cause a frequency shift. Another practical problem is that the sensor is validated by using a protected 3M transparent water-soluble wave solder tape 5414 Poly-vinyl alcohol (PVA) film and then drops of water at the centre of the resonator, which may give inconsistent responses of the tested water since the water drops float at different points unlike using a tube or microfluidic channels.

**Figure 12:**
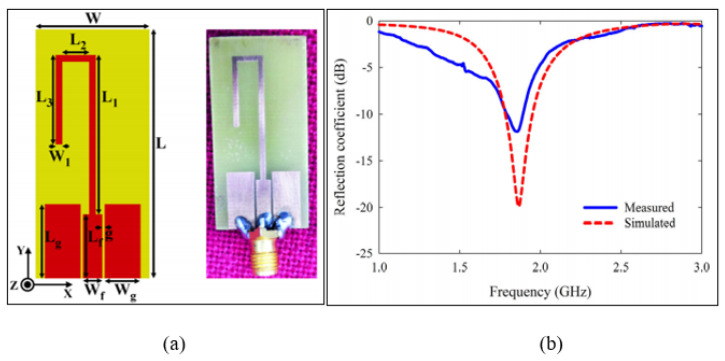
The coplanar waveguide fed open stub resonator sensor: (**a**) Layout of the CPW fed open stub resonator where the length and width are 38 mm and 17.8 mm, respectively. (**b**) Measured and simulated responses in the unloaded condition [79]. Reprinted from Moolat, R. et al. Liquid Permittivity Sensing Using Planar Open Stub Resonator. *Journal of Electronic Materials*, 2020, with permission from Springer Nature.

Another type of coplanar waveguide was proposed by Moolat et al. [79], which was based on a quarter-wavelength technique. The open stub resonator was double folded to minimize the circuit size, and the sensor was designed with a single port to operate at a 1.8 GHz resonance frequency. Figure 12 demonstrates the coplanar waveguide fed open stub resonator. This study provided a new insight into material characterization by immersing the sensor into the testing liquid samples. However, a large amount of liquid sample was required in order to perform the testing, which was a volume of 30 mL. Another issue is that it was limited to a certain range of the complex permittivity between 23.1 and 32.7 for the real part and 0.02 to 0.2 for the imaginary part. It is better for this type of resonator to have a protective adhesive film since the sensor will be affected by the contact tested chemical materials, which will reduce the sensitivity and performance of the sensor’s measurements.

**Figure 13:**
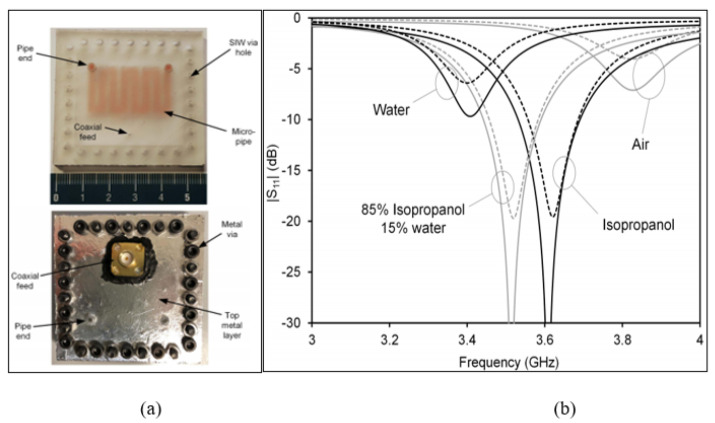
The Substrate-Integrated Waveguide (SIW) sensor: (**a**) Layout of the SIW sensor along with embedded micropipe. (**b**) Measured and simulated reflection response for empty (air), pure isopropanol, a mixture of isopropanol and water, and distilled water. The solid and dashed lines represent the simulation and measurement results, respectively [80].

Rocco et al. [80] conducted another technique for a cavity resonator based on Substrate-Integrated Waveguide (SIW) technology. The SIW sensor was developed based on a square cavity structure and a 3D printed and embedded multi-folded pipe, named as the inner meander pipe, with an inlet and outlet for injecting liquid samples, and it was fed by a coaxial probe. Although the 3D printing material had large dielectric losses, which degraded the quality factor, the authors minimized the dielectric filling of the cavity and also minimized the thickness of the pipe walls. This was to avoid a large loss in the cavity, which affected the loss tangent of the sample. Figure 13 illustrates the design of the substrate-integrated waveguide along with the 3D printed microfluidic channel for inlet and outlet liquid flow. It was used to test materials with a high range of permittivity from four to 76 at an operating frequency of 3.82 GHz. The SIW technology in this study used a metal via that produced a high electric field to increase the sensitivity; however, this increased the cost of the fabrication and measurement due to the design complexity.

**Figure 14:**
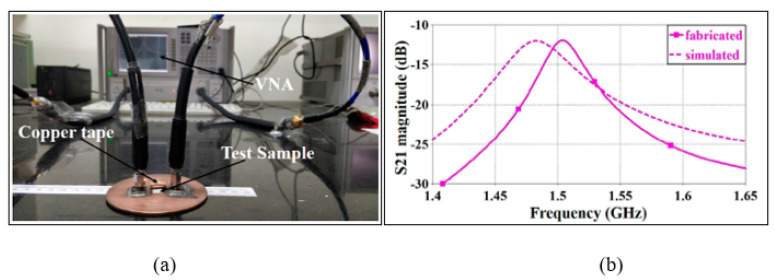
Cylindrical cavity resonator sensor: (**a**) Fabricated cavity-based cylindrical resonator along with the tested sample. (**b**) Measured and simulated transmission responses [84]. Reprinted from Varshney, P.K.; Akhtar, M.J. A compact planar cylindrical resonant RF sensor for the characterization of dielectric samples. *Journal of Electromagnetic Waves and Applications*, 2019, with permission from Taylor and Francis.

Moreover, the SIW sensors have various structure designs such as rectangular and circular. A comparison between these structures was discussed by Varshney and Akhtar [84]. The authors designed a cylindrical SIW cavity at a 1.5 GHz operating frequency and then compared the findings with a rectangular SIW cavity by considering the same parameters. The developed cylindrical SIW sensor exhibited an increase in sensitivity by 25% and minimized the overall circuit size by 22% compared to a rectangular SIW sensor. Figure 14 demonstrates the cylindrical cavity resonator sensor along with the loaded sample. The cavity resonator was designed in fundamental mode $TM_{010}$ at a 1.5 GHz operating frequency and used for detecting solid samples. Cavity resonators usually produce a high electric field for sensitivity and accuracy measurement; however, in this case, it produced 4.524 × $10^{3}$ v/m, which is quite low for cavity resonators.

**Figure 15:**
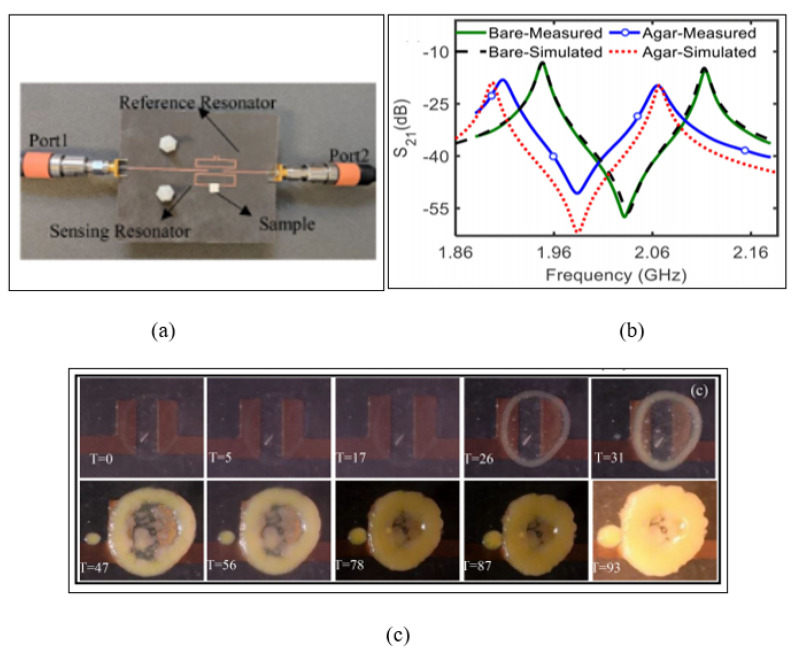
Microwave sensor based on a pair of split ring resonators: (**a**) Layout of the microwave sensor along with the two split resonators used as reference and sensing resonators. (**b**) Measured and simulated transmission response for the bare and loaded sensor. (**c**) Captured time-lapsefor the growth of bacteria associated with the experiment where T is time in hours [81]. Reprinted from Mohammadi, S. A Label-Free, Non-Intrusive, and Rapid Monitoring of Bacterial Growth on Solid Medium Using Microwave Biosensor. *IEEE Transactions on Biomedical Circuits and Systems*, 2020, with permission from IEEE.

While many resonator sensors focus on the detection of solid and liquid materials, resonator sensors also have applications in monitoring bacterial growth on solid agar media. A non-invasive and real-time microwave biosensor based on a pair of planar split ring resonators was proposed by Mohammadi et al. [81], which can monitor the growth of bacteria in a solid medium. Two planar SRRs were used to design the structure of the sensor, operating at two resonance frequencies for differential operation at 1.95 and 2.11 GHz. The reason for using two SRRs was to eradicate the noise of common electronics and the impact of permittivity variation because of humidity and ambient temperature on the sensor. One of the SRRs represents the sensor, which in this case detected the bacterial growth, and the other one was used as the reference signal to monitor the effects of the surrounding environment. Figure 15 illustrates the microwave sensor based on split ring resonators where the resonator with a slightly smaller physical size was designed to operate as the reference resonator and the larger one as the sensing resonator. To monitor the growth of the bacteria, a Petri dish was placed over the sensor covering both SRRs. Then, Luria–Bertani (LB) agar with a 4 mm thickness was inoculated by spreading Escherichia coli (*E. coli*) of 50 L over the sensing resonator while the other resonator was used as the reference. Figure 15c demonstrates the growth of the *E. coli* on the LB agar associated with the experiment, which was captured at different times. The sensor exhibited a good performance with the capability of monitoring the growth of bacteria in solid medium with a low concentration of 1.8 × 10^7^ cells/cm^2^.

**Figure 16:**
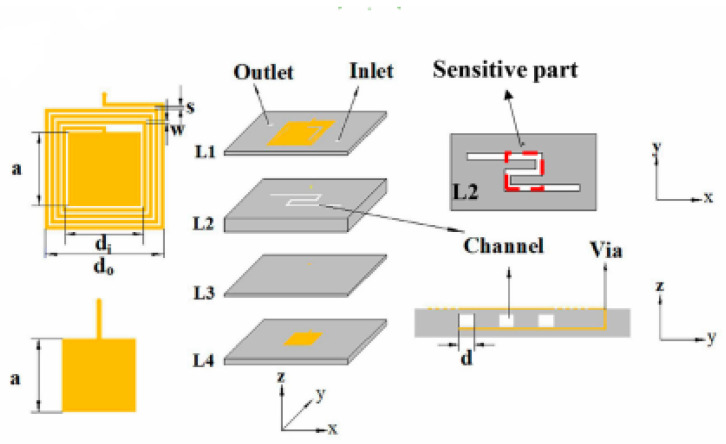
Design of a wireless LC microfluidic sensor using the Low Temperature Co-fired Ceramic (LTCC) multilayer technology [179].

Compared to current reported microfluidic planar resonator sensor technologies based on PCB or polymer substrate materials integrated with PDMS or Teflon as a fluidic channel, Low Temperature Co-fired Ceramic (LTCC) technology can be used for lab-on-a-chip and biosensing due to the easy integration with microfluidic devices and electronic circuits in a ceramic substrate. LTCC technology is based on multiple layers, and it can be fabricated using laser micromachining, mechanical milling or hot embossing [179,180]. Liang et al. [179] introduced a wireless LC microfluidic sensor based on LTCC technology. A planar spiral inductor along with a parallel plate capacitor (LC) resonant antenna were used to design the sensor, and then, it was integrated with microchannel bends in the LTCC substrate. Figure 16 illustrates the design of the wireless LC microfluidic sensor using the LTCC multilayer ceramic process. The LTCC can be a promising material for the development of microfluidic applications due to its ease of integration. However, the LCTT manufacturing process involves sintering at very high temperatures of 800–900 °C, resulting in a high cost and difficult fabrication.

## 5. Discussion and Research Challenges

### 5.1. Quality Factor Analysis

The Quality factor (Q-factor) is an essential parameter for estimating the quality of microwave sensing resonators. The unloaded Q-value ($Q_{0}$) means the Q-value for the microwave resonator without loaded the MUT, which is relevant to the conductivity of metal materials, as well as the filling materials within the cavity such as waveguide and coaxial resonators or above and inside the substrate such as planar resonators. The unloaded Q-factor can be calculated as [36,85,86]:

(12) $$Q=\frac{2f_{o}}{\Delta f}$$

where *Q* is the quality factor, *f* is the resonance frequency, and $\Delta_{f}$ is the bandwidth of the frequency ±3 dB with respect to minimal transitions. The loaded Q-factor can be expressed as described in [85,87]:

(13) $$Q=\frac{f_{o}}{\Delta f}$$

**Figure 17:**
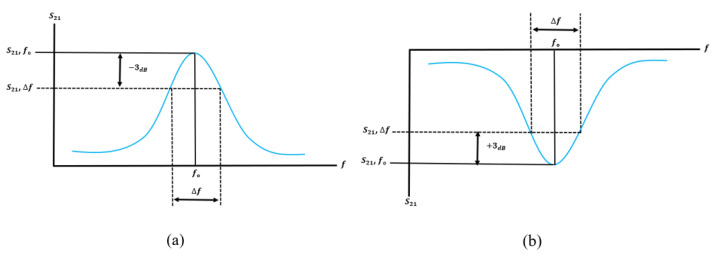
Measurement of the quality factor from S21: (**a**) Bandpass response. (**b**) Bandstop response.

The Q-factor increases as the shift in resonance frequency decreases. Thus, a narrower band and a sharper dip of the cut-off frequency of the bandwidth lead to higher Q-factor value. Figure 17 demonstrates the measurement of the quality factor obtained from the scattering parameter S21 based on the bandpass [36,85,86] and bandstop responses [16,76,88,89,90,91].

### 5.2. Sensitivity Analysis

To quantify the sensitivity of the resonator sensor, the resonance frequency for the empty case and for the case of being filled or loaded with the MUT are considered. The empty sensor is usually used as the reference model since the relative permittivity and the loss tangent of air are well known. When the sensor is loaded with the MUT in the strongest generated area of the electric field, the resonance frequency is changed, which is fully dependent on the permittivity of the tested materials. This relation is linear where any changes in relative permittivity ($\Delta \epsilon_{r}$) cause a change in the resonance frequency ($\Delta f_{r}$). Therefore, the sensitivity *S* of the sensor can be found from [12,74,92,93,94]:

(14) $$S=\frac{\Delta f}{\Delta \epsilon }=\frac{f_{empty}-f_{\epsilon_{r}}}{\epsilon_{r}-1}$$

where $\epsilon_{r}$ is the permittivity of the loaded sample, $f_{empty}$ is for the resonance frequency of the empty sensor and $f_{\epsilon_{r}}$ is the resonance frequency of the loaded sample.

For a fair comparison of the sensitivity, the sensitivity must be normalized to the resonance frequency of the empty sensor. This is due to sensors designed at high frequencies having a large absolute variation of the notch frequency with respect to the dielectric constant. The normalized sensitivity can be found from [10,12,18,23,80,95,96,97]:

(15) $$S\left(\%\right)=\frac{f_{empty}-f_{\epsilon_{r}}}{f_{empty}\left(\epsilon_{r}-1\right)}\times 100$$

where $f_{empty}$ is the resonance frequency of the empty sensor, $f_{\epsilon_{r}}$ is the resonance frequency of the loaded sample, $\epsilon_{r}$ is the dielectric constant of the loaded sample and S(%) is the normalized sensitivity.

### 5.3. Possible Location of the Material under Test

**Figure 18:**
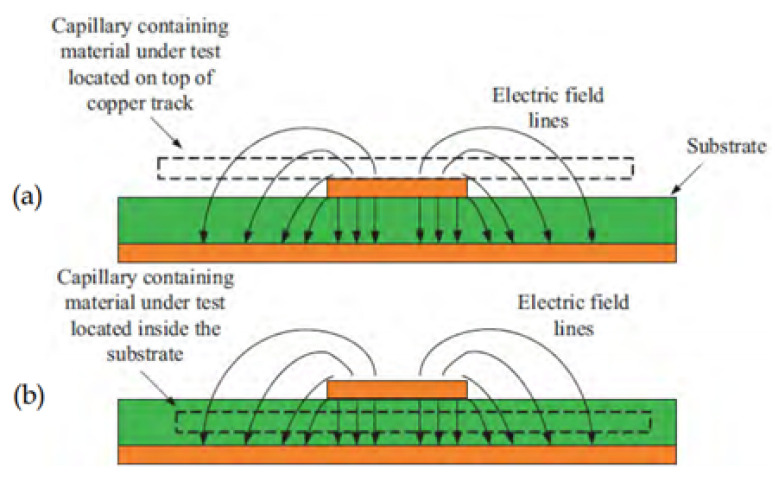
The possible location of the MUT: (**a**) Above the copper track: (**b**) Inside the substrate [98].

Usually, the properties of materials can be characterized using planar resonator sensors in the selected modes, and each of the considered measuring resonators is designed to ensure the best excitation conditions for necessary azimuthally symmetrical Transverse Electric (TE) or Transverse Magnetic (TM) modes [181]. The absolute values of the maximum field occur in fundamental mode (first resonant frequency) compared to the other modes. The response behaviour of the sensor is totally dependent on the interaction of the loaded MUT and the electric field distribution. The tested materials can be located either on top of the copper track or inside the substrate. The sensitivity and accuracy of the measurement is dependent on the extent of field penetration inside the tested material. Thus, the MUT must be located where the maximum Electric field (E-field) occurs. Figure 18 demonstrates the possible location of the MUT for material measurement. It can be seen that the electric field could be more concentrated in the top or inside the substrate in the design. For this reason, it is obvious that this results in a high sensitivity of the permittivity variation produced by locating the material under test in the maximum electric field (above the copper track or inside the substrate) [98].

### 5.4. Open Challenges

Microwave planar resonators have been extensively reported in recent years due to their advantages such as the ease of fabrication, low cost, and small physical size, which are compatible with many applications. However, these kinds of approaches carry with them various well-known limitations such as low quality factors (Q-factor), sensitivity, performance, and electric fields, which restrict their application in materials’ characterization. One of the serious limitations with this explanation is that the normalized sensitivity is quite low in detecting the properties of the materials using the planar resonators. Table 5 demonstrates recently reported work on microwave planar resonators in detecting the materials’ properties in terms of the complex permittivity (real and imaginary parts). It also shows the detection range of the complex permittivity for each designed sensor aligned with the resonance frequency for a bare sensor and the unloaded, and loaded conditions. It can be illustrated that the maximum normalized sensitivity was achieved by [11,12,75,95,99]. As reported by Ebrahimi, Scott and Ghorbani [12], the sensitivity was enhanced by incorporating one capacitor in the resonator sensor, while Abdolrazzaghi, Daneshmand and Iyer [95] substituted the conventional microwave couplers with metamaterial-based couplers to enhance the sensitivity. This indicates that those sensors have the capability to detect the properties of materials in a wider range of complex permittivity. However, low values of normalized sensitivity have serious limitations for many recently reported studies, as shown in Table 5 [15,16,45,55,59,100,101,102,103]. This might be due to producing a low Q-factor since they are planar structures and distribute the electric field along the large structure of the sensor, which reduces the interaction between the tested materials and the electric field of the sensor. These factors therefore need to be enhanced to make the sensor highly sensitive for a small volume of a binary mixture or concentration detection. On the other hand, Table 6 demonstrates various studies for detecting the concentration between the glucose dissolved in water solutions. For a reasonable comparison, the resonance frequency response of the channel filled with DI water is used for the standard comparison in the liquid under test case. It can be illustrated from the data in Table 6 that [65,104] reported significantly more normalized sensitivity than other studies [68,83,105,106,107,108]. This is due to there being no channel in sensors reported by [65,104] for controlling the fluidics and volume of the liquid sample, which potentially increases the cross-sensitivity of the sensor.

A variety of microwave planar resonator techniques are used to detect and characterize the properties of materials in terms of complex permittivity. Each has its advantages and drawbacks. A major advantage of these techniques is that they have a simple design and fabrication along with minimizing the circuit size. However, there are certain drawbacks associated with the use of these techniques such as low electric field confinement, Q-factor, and sensitivity, which restrict their use in many applications and limit the range of permittivity detection. To enhance the sensitivity, the electric field must be highly confined on and around the sensor so that most of the energy will be concentrated on desired area [117]. Table 7 demonstrates the recent designs of microwave resonators aligned with the electric field profile. As can be seen from Table 7, most of the designs have a low field confinement on and around the sensing area, thereby making the area slightly sensitive for the effective sensing of liquid concentration and binary mixtures. Therefore, a high electric field confinement on and around the sensing area is required to achieve high sensitivity in detecting a binary liquid mixture precisely.

Furthermore, a summary of the physical and electrical size for the recently reported sensors in previous works aligned with sensing and measurement types is presented in Table 8. All the microwave planar resonator studies reviewed so far, however, suffer from the fact that the measurements have only limited applications. Table 9 indicates various designs of microwave planar resonators aligned with the measured application of material characterizations. The transmission and reflection measurement techniques are considered in this comparison. Most of the researchers focused on liquid materials’ detection along with their binary mixtures or concentrations [82,95,118,133,134,135,136], while others focused on certain applications such as solid material detection [93,137,138], aqueous glucose detection [10,83,134,139] and food detection [74].

### 5.5. Research Directions

The topic of sensing the presence of a substance in an environment, whether the substance is solid, liquid or a binary mixture, is one of the most active areas in materials science research today. This is due to the increasing demand of important industrial applications such as quality control in material science, in the food industry and biomedical sensing [43,64,152]. Electrical characteristics, which are dependent on the dielectric properties of the materials, are inherent in every material. Thus, accurate determination of these properties helps engineers and scientists to put the material to proper use for more solid designs, moisture content measurement and improved quality control for various manufacturing processes from construction materials to pharmaceuticals. One of the main obstacles, as an example in biomedical applications, is the ability to monitor the condition of diabetes and detect the blood glucose level at the earliest possible stage, which is of significant interest in many engineering disciplines. Currently, the most widely used glucose detection methods rely on invasive testing, whether hospital/clinic health tests or localized testing techniques. These methods require a small drop of blood to be applied onto a portable blood glucose test strip. Then, the strip is inserted into measurement devices for reading the level of the blood glucose [153]. However, with the increasing number of diabetes patients, which is over 463 million affected people worldwide [154], the potential for blood glucose detection is too great to be economically tested using conventional and portable blood glucose techniques where the test is repeated up to 10 times daily for patient depending on the severity of diabetes, which causes discomfort. Moreover, the contamination of blood is considerable, where the test strip and needle must be disposed of to avoid blood-related diseases [153]. Therefore, a non-invasive measurement device is highly desirable to avoid the discomfort and aforementioned risks [153,155,156,157]. Microwave sensors at radio frequency have the potential to measure the change in the blood glucose dielectric properties [153,158] where a range from 72 mg/dL to 108 mg/dL is considered as the normal glucose level in blood. In the case of patient experiencing diabetes with a high glucose level, such as 400 mg/dL, it is required to regulate it with medication or insulin to reduce the chance of long-term negative health effects [159]. Further investigation and experimentation into a non-invasive pain-free glucose monitoring using microwave planar sensors are strongly recommended.

Another closely related obstacle to food industry application is the moisture content, which affects the products’ quality in various ways. Some are prone to fungal, bacterial and pest contamination, while others result in improper processing in an undesirable high moisture content environment. This leads to reducing the quality, efficacy and storage life of foodstuffs, drugs and chemicals and even poses a significant risk through food poisoning. Furthermore, the originality of food composition is widely considered as a main factor [160,161]. The presence of uncertain ingredients in some frozen or junk foods, as examples, needs to be known to recognize some allergies or diseases [162,163,164]. The metabolism or microbial growth over time for the stored food in refrigerators requires accurate understanding of the combination of its ingredients. One of the strengths of the reported study presented by Varshney, Sharma and Akhtar [74] is that the microwave planar resonator sensor has the capability of exploring the adulteration of some food materials. Therefore, it is suggested that the association of food material factors be investigated in future studies using microwave planar resonator sensors.

Another important aspect is quality control, such as water content [165]. For instance, water is under constant monitoring for the level of harmful suspensions in refineries. These, and more alternative examples, are the daily practice of different industries for maintaining the production level in a healthy state and controlling the incoming inspections of production with appropriate knowledge of the process. Highly accurate measurement sensors with consistently good quality measurement are required to monitor the small variation among different material samples. The prevailing sensing devices are expensive to install, along with their labour intensive performance for widespread inspection, which are considered as limiting factors affecting the quality of the outcome. Therefore, the development of microwave planar sensors that can offer the same performance with low cost production is required for application in industrial conditions.

A variety of microwave planar sensors are used for material characterization and their constituents in order to be used for monitoring in the food industry and for biomedical and quality control purposes [44,165,166]. Each has its advantages of having a high accuracy measurement, high sensitivity and fast detection [167,168] and the drawbacks of a bulky design, high fabricating cost and requiring a large amount of the sample for the measuring process [44,169,170,171,172]. Among various planar resonators, the split ring resonators (SRRs) and complementary split ring resonators (CSRRs) can be considered as the best suited for the realization of the resonant structure. The advantages of these resonators lie in their very small electric size, especially for the lower microwave band [9,11,34,36,42,54]. The size compactness, cost effectiveness and ease of fabrication are the key factors of planar sensors. However, a low Q-factor and sensitivity are the limiting factors in many important applications, which limits the range of material detection [24,34,171,173,174]. Therefore, future studies need to be carried out in order to enhance these limiting factors of microwave planar resonator sensors.

To enhance the sensitivity, the authors in [12] did so by incorporating one capacitor in the resonator sensor, while Abdolrazzaghi, Daneshmand and Iyer [95] substituted the conventional microwave couplers with metamaterial-based couplers to enhance the sensitivity. This indicates that those sensors have the capability to detect the properties of materials in a wider range of complex permittivity. However, low values of normalized sensitivity pose serious limitations for many recently reported studies, as reported in [15,16,45,55,59,100,101,102,103]. This might be due to producing a low Q-factor since they are planar structures and distributing the electric field along the large structure of the sensor, which reduces the interaction between the tested materials and the electric field of the sensor. The electric field must be highly confined on and around the sensor so that most of the energy will be mostly concentrated on the desired area [117]. These factors therefore need to be enhanced in the future studies to make the sensor highly sensitive with high electric field confinement on and around the sensing area for a small volume of a binary mixture or concentration detection.

Another important key restrict factor is the limited measurement applications based on microwave planar resonators. Most of the researchers focused on the liquid materials’ detection along with their binary mixtures or concentrations [82,95,118,133,134,135,136], while others focused on certain applications such as solid material detection [93,137,138], aqueous glucose detection [10,83,134,139] and food detection [74]. Therefore, there is the desire for novel sensors that could be applied in various measurement applications and offer an enhanced performance with lower production cost. Further research should be undertaken to explore how microwave planar sensors can be used in a large number of possible applications.

## 6. Conclusions

The aim of this paper is to review recent research into RF planar resonator sensors, mainly focused on passive resonators. The study critically addresses the low normalized sensitivity as the main challenge related to planar resonator sensors for material characterization and identifies research directions to motivate and facilitate researchers to contribute to this topic. The techniques of extracting complex permittivity (real and imaginary parts) are discussed based on the different approaches of the mathematical models. The observations from this study suggest that the complex permittivity extraction based on ANNs provides high accuracy and reliable performance in a short time compared to empirical models. Further research might explore how to achieve high normalized sensitivity since the reported normalized sensitivity is within the range of 0.026% and 0.43% for planar resonator sensors. This study clarifies the recent development of material characterization techniques based on planar resonator sensors and provides current limitations for researchers who are interested in microwave resonators for material characterization.

## Figures and Tables

**Table 1:** Comparisons of the most popular techniques and their advantages and disadvantages. NRW, Nicholson–Ross–Weir; RFM, Rational Function Model.

Measurement Techniques	Advantages	Disadvantages	Materials Under Test	Conversion Methods	Speed	Accuracy
Transmission and reflection line	- Used to measure samples with medium to high loss - Used to determine both permittivity and permeability	- Limitation of measurement accuracy of the air-gap effects - Low accuracy for a sample whose length is a multiple of one-half-wavelength in the materials	- Solid - Liquid - Sheet surface	- NRW - NIST iterative - New non-iterative	Fast Slow Fast	Medium Good Good
Open-ended coaxial probe	- Easy sample preparation - Measurement for the large number of samples in a short time after the calibration - Measurement can be performed in a temperature-controlled environment	- Supports only reflection measurement - Affected by the air-gap for measurement in the specimen	- Liquids - Biological specimens - Semi-solids	RFM	Fast	Good
Free space	- Suitable for high-frequency measurement - Allows non-destructive measurement - Measures material under test in hostile conditions - Evaluates both permittivity and permeability properties	- Needs large and flat material under test - Multiple reflections between the surface of the sample and the antenna - Diffraction effects at the edge of the sample	- High temperature materials - Large flat solid - Gas - Hot liquid	- NIST iterative - New non iterative - NRW	Slow Fast Fast	Good Good Medium
Resonant methods	- Suitable to measure small materials under test - Uses approximate expressions for the field in both the sample and cavity	- Requires the high-frequency resolution Vector Network Analyser (VNA) - Limited to only narrow frequency bands	- Rod shaped solid materials - Gas - Liquids	Frequency and quality factors	Slow	Good

**Table 2:** Selected published articles that detected and characterised various solid materials. MUT, Material Under Test; IDC, Interdigital Capacitor; CSRR, Complementary Split Ring Resonator.

Ref.	Technique	Substrate Material *	Freq (GHz)	Size (mm)	Tested Material (MUT)	Remark
[39]	Splitter/combiner microstrip with IDCs and 2 CSRR structures	Fabricated on Rogers5880 $\epsilon_{r}$ = 2.20 *h* = 1 mm *t* = 0.018 mm	5.6	N/A	Viz, PVC, glass epoxy, FR4, glass	The measured complex permittivity of the test specimens is found to be in close agreement with their values available in the literature with a maximum error of ≤5%. Disadvantages: Low electric field distribution (2.667 × $10^{3}$) v/m
[33]	Metamaterial SRR	Fabricated on FR4glass epoxy board $\epsilon_{r}$ = 3.583 *h* = 1.5 mm	3.57	8 × 8	Calibration: plastic, Perspex, glass epoxy, glass Extracted: Rice flour, wheat flour, all-purpose flour, corn flour, green gram flour, gram flour, soya flour	Advantages: - Potential application in the field of food preservation and quality checking - Detecting real permittivity Disadvantage: - Low permittivity detection - Low accuracy - No weighing of flour samples - Loss tangent not determined
[34]	SRR	Fabricated on RT/duroid 6006 *h* = 1.27-mm	2.5	80 × 40	Viz, Teflon, polyvinyl chloride, Plexiglas, polyethylene, carbonyl iron, Ni${}_{0.6}$Co${}_{0.4}$Fe${}_{2}$O${}_{4}$, cobalt, 30% polystyrene composite	The measured relative permeability and the relative permittivity of the test specimens are found to be in close agreement with their values available in literature with a maximum error of less than 8%.
[46]	Two spiral inductors and IDC	Fabricated on FR4 *h* = 1.6 mm	2.2–2.8	44 × 24	Magnetic, soft cobalt steel (SAE 1018), ferrite core rubber, plastic, wood, white marble	Experimentally, it is found that complex permeability and permittivity measurement is possible with an average error of 2%.
[178]	A modified ring resonator	Fabricated on RT/duroid 5880 $\epsilon_{r}$ = 2.2 *h* = 1.58 mm tanδ = 0.001	3.2	28 × 4	Peat soil, sand soil	The model has within a 5% error in dielectric estimation with the commercial dielectric probe for peat soil (≥28% m.c.) and sand soil (≥10% m.c.).
[38]	A metamaterial planar sensor	Fabricated on FR4 *h* = 0.8 mm	5.3–8.2	30 × 30	Benzene, ethyl acetate, hexane, N-pentane, polyethylene, PVC, Teflon, THF	For solid and hazardous liquid samples. Disadvantages: - Limited permittivity detection from 1 to 10 real permittivity.
[25]	Microstrip ring resonator	Fabricated on Roger5880 $\epsilon_{r}$ = 2.2 *h* = 787 um tanδ = 0.0009 *t* = 17.5 um	1	141 × 87.5	Air, meat	The maximum discrepancy is about 6% only. Disadvantages: - Simulated and measured results have a discrepancy, which is observed for the loss magnitude (around 15%).

* *h* = thickness, *t* = copper thickness, $\epsilon_{r}$ = dielectric constant, and tanδ = loss tangent. N/A = Not Available.

**Table 3:** Various liquids’ detection based on existing studies found in the literature.

Ref.	Technique	Substrate Material *	Freq (GHz)	Size (mm)	Tested Material (MUT)	Remark
[48]	A transmission/reflection (TR) measurement scheme	RT/duroid-5880 *h* = 0.8 mm	8 to 12	22.86 × 10.16	Clear oil, dark oil, gasoline, ethanol, butanol	In this setup, different error sources can reduce the precision of the characterization; for example, a slight change in the sample position within the waveguide across the FSS aperture can cause measurement errors.
[51]	A simple microwave resonant sensor	FR4 $\epsilon_{r}$ = 4.3 tanδ = 0.025.	5.85	34.7 × 24	Petrol, water	The measured results show that the proposed sensor is capable of detecting up to 5% of petrol in water.
[4]	VSRR	RT/duroid-5880 $\epsilon_{r}$ =2.2, *h* = 1.57 mm	2.45	53.2 × 53.2	N-hexen, petrol, diesel	Error is within 1.53%. Sensitivity is 13.33 MHz per 1% change in real permittivity. Disadvantages: The petrol sample was varied in a range from 0 to 6%; limited to low permittivity detection.
[52]	Microstrip slot antenna	FR4 *h* = 1 mm $\epsilon_{r}$ = 4.4	2.4	N/A	Air, olive oil	The suitable permittivity measurement range for the proposed sensor has a relative error of 3.6%.
[47]	Planar RF sensor	FR4 *h* = 1.6 mm	5.85	34.7 × 24	Olive oil, coconut oil, soya bean oil, sunflower oil, mustard oil	A planar resonant RF sensor based on a generalized IDC-like structure is used for liquid characterization.

${}^{*}$h = thickness, *t* = copper thickness, $\epsilon_{r}$ = dielectric constant, and tanδ = loss tangent. N/A = Not Available.

**Table 4:** Various liquids’ mixtures detection based on existing studies found in the literature.

Ref.	Technique	Substrate Material *	Freq (GHz)	Size (mm)	Tested Material (MUT)	Remark
[68]	ENGResonator	FR-4 $\epsilon_{r}$ = 4.4 *h* = 1.6 mm	2.074	40 × 20	Water-glucose	The concentration range of the glucose is 20–100 mg/mL.
[67]	RF patch biosensor	N/A	17.25 and 21.09	3.3 × 2	DI water, D-glucose powder	The concentrations range of the glucose is 50–600 mg/dL. The sensitivity achieved is up to 1.13 MHz and 1.97 MHz per mg/dL, and the detection limits are 26.54 mg/dL and 15.22 mg/dL.
[70]	Broadband dielectric spectroscopy	N/A	2 to 18	N/A	N/A	The concentration is varied from 0 to 10 mg/mL. The sensitivity achieved is 0.15 dB/(mg/mL) and 0.5°/(mg/mL) at a room temperature of 25 °C.
[69]	Antenna-coupled SRR	N/A	1.9	8.3	N/A	The tested material is blood plasma with different glucose concentrations.
[71]	Antenna-driven ring	FR4 *t* = 35 um	2	30 × 30	DI water, glucose, NaCl solutions	A 17.5 MHz shifting of the resonance frequency is obtained with a high rate of error of 7.3%. The sensitivity achieved is 0.107 MHz/mg dL${}^{-1}$.
[72]	Transmission/reflection line method	N/A	1 to 1.20	N/A	N/A	The tested range of water-glucose concentration is from 50 mg/dL to 1000 mg/dL with a frequency range from 100 MHz to 4 GHz.
[66]	Planar MRR	FR4 Epoxy $\epsilon_{r}$ = 4.4	1	108.75 × 65	Glucose percentage	The concentration of water-glucose is tested, and there is a slightly frequency shift. However, the sample is directly tested on the sensor, which increases the measurement error.
[65]	RMLresonator	N/A	9.20	2 × 0.854	Glucose percentage	The sensitivity achieved is 1.08 MHz per 1 mg dL${}^{-1}$. The detection limit is 8.01 mg dL${}^{-1}$ with a quantisation limit of 24.30 mg dL${}^{-1}$.

* *h* = thickness, *t* = copper thickness, $\epsilon_{r}$ = dielectric constant, and tanδ = loss tangent. N/A = Not Available.

**Table 5:** Comparison of recently reported work on microwave planar resonators in detecting the material properties in terms of sensitivity, detection range and complex permittivity.

Ref.	Source Title	Sensor	Sample Containers	Bare Sensor $f_{o}$ (GHz)	$f_{\mathrm{empty}}$ (GHz)	$f_{\mathrm{water}}$ (GHz)	Sensitivity (MHz/Δϵ)	Normalized Sensitivity (%)	Detection Range ($\epsilon^{\prime }$/$\epsilon^{\prime \prime }$)	Complex Permittivity
[16]	Sensors Actuators A: Physical	SRR	PET film microfluidic channel	2.1	2	1.85	1.83	0.091	9–83/6–15	Real/imaginary
[11]	IEEE Sensors Journal	CSRR	PDMS microfluidic channel	2.4	2	1.52	6.11	0.31	9–79.5/9–15.6	Real/imaginary
[55]	IEEE Trans. Microw. Theory Techn.,	SRR	Capillary microfluidic	≈3	3.1035	3.042	0.805	0.026	1.893–77.42/0.001–12.49	Real/imaginary
[109]	Sensors, 2016	SIW	PDMS microfluidic channel	18	17.08	14.95	27.31	0.16	2.3–79/-	Real
[110]	IEEE trans.antennas propagation	SIW	Microfluidic channel (etched on the PMMA substrate using a laser)	5.06	4.69	4.2	6.24	0.13	-	-
[45]	Microw. Opt. Tech. Lett.	Double SRR	Micro-capillary	2.1	2.1213	2.0402	1.03	0.048	24.49–80.1/-	Real
[111]	J. Applied Physics	Dual-gap SRR	PDMS microfluidic channel	3.24	3.23	2.98	3.27	0.1	8–77.5/8.8–16.6	Real/imaginary
[59]	IEEE Microw. Wirel. Compon. Lett	Bridge SRR	Micro-capillary	2.35	2.27034	2.20220	0.861	0.038	24.5–80.1/-	Real/-
[54]	IEEE Sensors Journal	Splitter/combiner SRRs	PDMS microfluidic channel	1	0.978	0.87	1.35	0.138	27.86–80.86/3.04–10	Real/Imaginary
[102]	J.Electrochemical Soc.	Single CSRR	Fluid	4.078	4.078	3.897	2.33	0.057	8.96–8.64/5.22–11.34	Real/imaginary
[103]	IEEE Microw. Wirel. Compon. Lett.,	SIW	Glass pipe	2.52	2.51	2.391	1.56	0.062	5.25–77.50/-	Real
[112]	IEEE Microw. Wirel. Compon. Lett.	PMR	3D printed microfluidic channels	5.8	5.77	5.31	5.86	0.1	9–79.5/-	Real
[99]	Sensors Actuators A	RCRR	Container	0.90	0.810	0.639	0.002	0.27	1–78.5/2.45–9.5	Real/imaginary
[113]	Sensors	Quarter-Mode SIW	PDMS channels	5.81	5.791	5.321	6.53	0.11	5.08–73/-	Real
[114]	Sensors	SIW	PDMS microfluidic channel	≈8	8.26	7.60	10	0.12	5–67/-	Real
[115]	Sensors	3D SRR	Teflon tube	2.56	2.559	2.350	2.79	0.11	6–76/-	Real
[15]	IEEE Sensors Journal	CSRR	Glass capillary	2.4	2.365	2.302	0.808	0.034	9–79/8.1–17.1	Real/imaginary
[95]	IEEE Trans. Microw. Theory Techn.	Microwave sensor	PTFE Tube	2.5	2.6	≈2.07	7.02	0.27	1–140/76.7	Real/loss tangent
[100]	Int. J. RF Microw. Computer-Aided Eng.	CSIWwith DMS	Micro-capillary channel	4.40	4.3392	4.1850	1.95	0.045	24.5–80.1	Real
[116]	IEEE Sensors Journal	Multi-layered mushroom HIS	Tube coiled within the sensor	4.455	4.340	4.018	4.18	0.096	1–78/0.00058–17.16	Real/imaginary
[101]	Scientific Reports	CSIW	Micro-capillary channel	4.4	4.4035	4.2510	1.93	0.044	24.5–80.1/0–22.99	Real
[12]	IEEE Trans. Microw. Theory Techn.	LC resonator	PDMS microfluidic channel	≈ 2.2	1.91	1.27	8.14	0.43	30–79.6/4–11.5	Real/imaginary
[75]	Measurement	SRR	Droplet on the sensor	5.7	5.76	4.80	12.5	0.22	24–78/-	Real
				7.8	7.85	6.35	19.74	0.251		
[80]	IEEE Trans. Microw. Theory Techn.	SIW	Embedded micropipe	≈3.82	3.8267	3.4077	5.62	0.15	4–75.6/	Real/loss tangent

**Table 6:** Comparison of various studies for detecting the concentration between the glucose dissolved in water solutions in terms of sensitivity and concentration range.

Ref.	Sensor	Sample Containers	Sensing Method	Bare Sensor $f_{o}$ (GHz)	$f_{0}$ mg/dL (GHz)	$f_{\max}$ (GHz)	Δf (MHz)	Sensitivity (MHz/mg·dL${}^{-1}$)	Normalized Sensitivity Per (mg·dL${}^{-1}$)	Concentration (mg/dL)
[105]	CE-LC resonator	Microfluidic PDMS channel	$f_{r}$	1.67	1.167	1.39	230 MHz	0.023	1.34 × $10^{-5}$	0–10,000 *
[83]	SRR with IDC	Microfluidic PDMS channel	$f_{r}$ & $S_{21}$	4.18	1.327	1.457	130	0.026	6.22 × $10^{-6}$	0–5000
[106]	RF/microwave single-port resonator	Non-invasive	$f_{r}$ & $S_{11}$	4.8	3.427	3.441	14	0.014	2.92 × $10^{-6}$	0–1000
[68]	ENG unit-cell resonator	PDMS cavity	$f_{r}$ & $S_{21}$	2.09	1.91	2.05	140	1.4/0.014	6.70 × $10^{-6}$	0–10,000 *
					1.42	1.59	170	1.7/0.017	8.13 × $10^{-6}$	
[107]	CSRR	PDMS microfluidic channel and PMMA frame	$f_{r}$ & $S_{11}$	2.6	2.48	2.5194	39.4	0.4925/0.00493	1.89 × $10^{-6}$	500–8000 *
[108]	Open loop resonator	Glass capillary	$f_{r}$ & Q	2	1.10929	1.10938	0.09	0.000023	1.13 × $10^{-8}$	0–4000
[104]	Mediator-free resonator	Droplet on the sensing area	$f_{r}$ & $S_{21}$	1.5	0.38	0.875	495	0.99	6.60 × 10${}^{-4}$	30–500 *
[65]	RML resonator with IPD	Droplet on the sensing area	$f_{r}$	9.2	9.14	8.68	460	0.92	1.00 × $10^{-4}$	25–500

* The concentration range is changed from mL to dL where 1 dL = 100 mL.

**Table 7:** Comparison of the recent designs of microwave resonators in terms of the electric field profile.

Ref.	Sensor	$f_{o}$ (GHz)	Applications	Electric Field (v/m)
[117]	Closed-loop enclosed SRR	6.1	For aqueous and blood-glucose measurements	1.225 × $10^{4}$
[118]	Circular CSRR	2.4	For water-ethanol mixtures	5.000 × $10^{3}$
[23]	Differential MCSRR	1.618	Liquid/PDMS	1.066 × $10^{3}$
[78]	Asymmetric CPW SRR	1.57	Solid and liquid sensing	5.000 × $10^{3}$
[119]	Metamaterial-based sensor	12-Aug	Branded and unbranded diesel	1.512 × $10^{4}$
[120]	Microwave planar resonator	4.5∼4.6	For monitoring organic contaminants in water	1.000 × $10^{4}$
[79]	CPW open stub resonator	1.8	Liquid permittivity sensing	1.130 × $10^{4}$
[82]	Dual-band using CSRR	2.45 & 5.8	For investigation of liquid impurity concentration	2.000 × $10^{4}$
[121]	Two CSRRs	2.36	For full characterization of magneto-dielectric materials	1.574 × $10^{3}$
[122]	SIW	2.2	Fully characterizing magneto-dielectric (MD) materials	1.905 × $10^{3}$
[123]	Symmetric CPW with IDC	4.5	For permittivity characterization	1.250 × $10^{4}$
[10]	Metamaterial-based microwave sensor	3.43	Liquid sensor	4.000 × $10^{4}$
[96]	Open CSRR	0.330–0.204	Dropping-based liquid dielectric characterization	1.800 × $10^{4}$
[124]	A sensor based on SSPPs	≈8.7	To detect defects in film-coated metals and non-metallic materials	1.000 × $10^{3}$
[101]	Circular SIW sensor	4.4	For aqueous dielectric detection	1.535 × $10^{4}$
[84]	Planar cylindrical resonant RF sensor	1.5	For the characterization of dielectric samples	4.524 × $10^{3}$
[125]	Microwave sensor based on an interdigital capacitor-shaped defect ground structure	1.5	For permittivity characterization	8.000 × $10^{3}$
[126]	Planar microwave probe	11.56	For adulteration detection in edible oils	8.295 × $10^{3}$
[127]	Radio frequency (RF) resonator-based noncontact sensor	1.37	Accurate quantification of uric acid in temperature-variant aqueous solutions	3.008 × $10^{3}$
[128]	SIW resonator	2.39	A binary water alcohol mixture	2.000 × $10^{4}$
[129]	Radio-frequency dielectric measurement	0.15	Determination of meat moisture content	1.312 × $10^{4}$
[130]	A resonator-based metamaterial	7.6	Solid materials	1.086 × $10^{4}$
[131]	Dual band planar microwave sensor	2.5 & 3.8	For dielectric characterization using solid and liquid samples	9.750 × $10^{3}$
[132]	Transmission line integrated metamaterial-based liquid sensor	8 to 12	Liquid materials	2.500 × $10^{3}$

**Table 8:** Summary of various designs of microwave planar resonators in terms of physical and electric size including sensing and measurement types.

Ref.	Sensor	*f* (GHz)	Fabricated Material Substrate	$\epsilon_{r}$	Physical Size (mm/$\lambda_{o}$)	Electrical Size ($\lambda_{g}$)	Structure	Sensing	Measurement Type
[18]	Square SIW	2.1887	Rogers4003	3.55	55 × 50/0.4 × 0.36	0.76 × 0.69	Planar	Liquid	Non-invasive and contactless
[140]	SIW	13.48	Rogers RT5870	2.33	33 × 28/1.48 × 1.26	2.26 × 1.92	Planar	Liquid	Non-invasive and contactless
[141]	SIW circular	2.6	F4B	2.65	36 × 34/0.3 × 0.29	0.51 × 0.48	Planar	Liquid	Non-invasive and contactless
[118]	CSRR	2.4	FR4	4.1	35 × 25/0.28 × 0.2	0.57 × 0.4	Planar	Liquid	Non-invasive and contactless
[23]	MCSRR	1.618	Roger RO4350	3.66	78 × 50/0.4 × 0.27	0.8 × 0.52	Planar	Liquid	Non-invasive and contactless
[142]	CSRR	5.39	Thin substrate	2.9	30 × 30/0.54 × 0.54	0.92 × 0.92	Planar	Solid	destructive and contact
[76]	CSSSR	15.17	FR4	4.4 *	30 × 25/1.52 × 1.26	3.18 × 2.65	Planar	Solid	Destructive and contact
[143]	MIMring resonator	3.82	Rogers RO4003	3.38	34 × 34/0.38 × 0.38	0.7 × 0.7	Planar	Liquid	Non-invasive and contactless
[144]	CSRR	3.1	FR-4	4.4	64 × 48/0.66 × 0.5	1.39 × 1.04	Planar	Solid/soil water content	Destructive and contact
[101]	Circular SIW	4.4	Roger RT5880	2.2	79.92 × 79.92/1.2 × 1.2	1.7 × 1.7	Planar	Liquid	Non-invasive and contactless
[145]	SIW with negative order resonance	2.5	RO4003C	3.55	50 × 30/0.42 × 0.25	0.79 × 0.47	Planar	Liquid	Non-invasive and contactless
[146]	Electrically-coupled resonators	2.5	Roger 5880	2.2	45.44 × 45.44/0.38 × 038	0.56 × 0.56	Planar	Solid	Destructive and contact
[147]	CSRR	2.9	Neltec NY9217(IM)	2.17	40 × 26/0.39 × 0.25	0.57 × 0.37	Planar	Liquid	Non-invasive and contactless
[148]	Double microstrip microfluidic sensor	2.61	Rogers 5870	2.33	52 × 42/0.45 × 0.37	0.69 × 0.56	Planar	Liquid	Non-invasive and contactless
[149]	Circular SIW	5	Rogers RT5880	2.2	75 × 75/1.25 × 1.25	1.85 × 1.85	Planar	Liquid	Non-invasive and contactless

* Assumed value.

**Table 9:** Comparison of various designs of microwave planar resonators in terms of measured applications in material characterizations.

					Types of Material Characterization				
Ref.	Source Title	Sensor	$f_{o}$ (GHz)	Technique	Aqueous Glucose Solution *	Solid	Pure Liquid	Binary Liquid Mixtures *	Food (Flours)
[133]	Sensors and Actuators, B: Chemical	Differential microwave sensor	≈2.45	Reflection	x	x			x
[118]	Sensors and Actuators, A: Physical	Circular CSRR	2.4	Transmission	x	x			x
[77]	IEEE Transactions on Microwave Theory and Techniques	Central gap ring resonator	2.5	Transmission	x	x		x	x
[137]	IEEE Transactions on Microwave Theory and Techniques	Coupled resonators	2.468 & 1.938	Reflection	x		x	x	x
[139]	International Journal of Microwave and Wireless Technologies	Flexible RF resonator with IDC	2.46	Transmission		x			x
[120]	Sensors and Actuators, A: Physical	Microwave planar resonator	4.5∼4.6	Transmission		x		x	x
[74]	International Journal of RF and Microwave Computer-Aided Engineering	Electric-LC resonator	3.9	Transmission	x		x	x	
[82]	Journal of Electronic Materials	Dual-band CSRR	2.45 & 5.8	Transmission	x	x			x
[107]	Sensors and Actuators, A: Physical	Microwave reflective	2.4–2.6	Reflection		x		x	x
[83]	IEEE Sensors Journal	Improved SRR	4.18	Transmission		x	x	x	x
[145]	IEEE Sensors Journal	SIW with negative order resonance	2.5	Reflection	x	x		x	x
[134]	IEEE Transactions on Instrumentation and Measurement	Open-loop resonator	1.92, 5.16, 7.16	Transmission		x			x
[150]	Sensors (Switzerland)	Complementary circular RR	≈2.4	Transmission	x	x			x
[135]	Scientific Reports	Microwave-microfluidic flow resonator	4	Transmission	x	x			x
[93]	IEEE Sensors Journal	Differential resonators with two SRRs	2.1	Transmission	x		x	x	x
[151]	Sensors (Switzerland)	Embedded microstrip line with CSRR	2.5	Transmission	x	x		x	x
[95]	IEEE Transactions on Microwave Theory and Techniques	Metamaterial coupling	2.5	Transmission	x	x			x
[136]	Sensors (Switzerland)	Re-entrant cavity	2.4	Transmission	x	x			x
[131]	Journal of Telecommunication, Electronic and Computer Engineering	Dual band planar microwave	2.5 & 3.8	Transmission	x			x	x

${}^{*}$ Aqueous glucose solution is: glucose-water concentration. Binary liquid mixtures are: glycerol-water solutions, water-ethanol, water-methanol, milk-urea solution.

## Data Availability

Not applicable.
